# Diagnosis of bronchiectasis and airway wall thickening in children with cystic fibrosis: Objective airway-artery quantification

**DOI:** 10.1007/s00330-017-4819-7

**Published:** 2017-05-18

**Authors:** Wieying Kuo, Marleen de Bruijne, Jens Petersen, Kazem Nasserinejad, Hadiye Ozturk, Yong Chen, Adria Perez-Rovira, Harm A. W. M. Tiddens

**Affiliations:** 1 Department of Pediatric Pulmonology and Allergology, Erasmus MC - Sophia Children’s Hospital, Gravendijkwal 230, 3015 CE Rotterdam, The Netherlands; 2 Department of Radiology, Erasmus MC, Rotterdam, The Netherlands; 3 Biomedical Imaging Group Rotterdam, Departments of Medical Informatics and Radiology, Erasmus MC, Rotterdam, The Netherlands; 4 Department of Computer Science, University of Copenhagen, Copenhagen, Denmark; 5 HOVON Data Center, Clinical Trial Center, Erasmus MC Cancer Institute, Rotterdam, The Netherlands; 6 Department of Biostatistics, Erasmus MC, Rotterdam, The Netherlands; 7 Department of Radiology, General Hospital of Ningxia Medical University, Yinchuan, China

**Keywords:** Cystic fibrosis, Imaging/CT, Paediatric lung disease, Bronchiectasis, Airway dimensions

## Abstract

**Objectives:**

To quantify airway and artery (AA)-dimensions in cystic fibrosis (CF) and control patients for objective CT diagnosis of bronchiectasis and airway wall thickness (AWT).

**Methods:**

Spirometer-guided inspiratory and expiratory CTs of 11 CF and 12 control patients were collected retrospectively. Airway pathways were annotated semi-automatically to reconstruct three-dimensional bronchial trees. All visible AA-pairs were measured perpendicular to the airway axis. Inner, outer and AWT (outer−inner) diameter were divided by the adjacent artery diameter to compute A_in_A-, A_out_A- and A_WT_A-ratios. AA-ratios were predicted using mixed-effects models including disease status, lung volume, gender, height and age as covariates.

**Results:**

Demographics did not differ significantly between cohorts. Mean AA-pairs CF: 299 inspiratory; 82 expiratory. Controls: 131 inspiratory; 58 expiratory. All ratios were significantly larger in inspiratory compared to expiratory CTs for both groups (p<0.001). A_out_A- and A_WT_A-ratios were larger in CF than in controls, independent of lung volume (p<0.01). Difference of A_out_A- and A_WT_A-ratios between patients with CF and controls increased significantly for every following airway generation (p<0.001).

**Conclusion:**

Diagnosis of bronchiectasis is highly dependent on lung volume and more reliably diagnosed using outer airway diameter. Difference in bronchiectasis and AWT severity between the two cohorts increased with each airway generation.

***Key points*:**

*• More peripheral airways are visible in CF patients compared to controls.*

*• Structural lung changes in CF patients are greater with each airway generation.*

*• Number of airways visualized on CT could quantify CF lung disease.*

*• For objective airway disease quantification on CT, lung volume standardization is required.*

**Electronic supplementary material:**

The online version of this article (doi:10.1007/s00330-017-4819-7) contains supplementary material, which is available to authorized users.

## Introduction

In cystic fibrosis (CF) lung disease is characterized by progressive bronchiectasis (BE) and airway wall thickening (AWT) [1–3]. Chest computed tomography (CT) is the most sensitive tool for diagnosing BE and AWT, which are important outcome measures for both clinical and research purposes [1, 4].

BE is defined as destructive and irreversible widening of airways with a ratio between airway and accompanying artery (AA) above 1 in adults [5, 6]. Currently, the inner airway dimensions are mostly used for comparison with the artery [7–9]. However, no clear consensus, based on objective quantitative measures, exists on whether inner or outer airway diameter should be compared with the artery for the diagnosis of BE [10]. Nonetheless, the approximate ratio of 1 was based on the outer airway diameter [11]. In addition, it is not clear whether identical AA-ratio cutoffs can be used to define BE in children, where a smaller AA-ratio has been suggested [5]. Lastly, the AA-ratio is thought to increase with age in healthy subjects [12].

AWT like BE is associated with airway inflammation [13, 14]. AWT is presumed to be present when the airway wall diameter occupies more than 20% of the total outer airway diameter [15] or takes up more than 33% of the adjacent arterial diameter [8]. CT assessments of BE and AWT are mostly performed in the axial or coronal plane by comparing airway diameter to the accompanying artery diameter. However, to avoid inaccuracy caused by the parallax or projection error, the AA dimensions should ideally be evaluated in a view perpendicular to the airway centre-line [16–18].

Airway dimensions are routinely evaluated on inspiratory CT [19]. Unfortunately lung volume levels during inspiratory CT acquisition have been shown to vary widely between 55% and 106% of the measured total lung capacity (TLC) obtained via body plethysmograph [20]. This variability is caused by a lack of breath-hold standardization and influences the AA-ratio and therefore diagnosis of bronchiectasis [7, 21, 22]. Airway dimensions assessed on axial view were shown to be highly dependent on the lung volume levels in children below the age of 5 years with CF [7], in adults with chronic obstructive pulmonary disease (COPD) [23, 24], and in healthy controls [22]. Hence, suboptimal lung volume levels negatively impact objective evaluation of BE and AWT.

The purpose of our study was to develop objective criteria to diagnosis BE and AWT in children by comparing AA dimensions between CF patients and control patients with spirometer-guided CTs. We hypothesized that AA-ratios are increased in paediatric CF patients and more prominent on inspiratory CTs. To investigate this, we aimed to assess: (1) AA dimensions in control patients; (2) use of inner or outer airway diameter as a more sensitive biomarker to diagnose BE; and (3) influence of inspiratory and expiratory CTs on AA dimensions. Between CF and control patients we aimed to assess: (4) differences in the number of visible AA-pairs and AA dimensions; and (5) differences according to airway location (e.g. lobes and airway generations).

## Materials and methods

This study was approved by the Institutional Review Board (MEC-2014-254). Written informed consent was waived for all patients because of the retrospective nature of the study.

## Study population

Spirometer-guided inspiratory and expiratory chest CTs of 11 CF patients and 12 control patients without lung abnormalities on CT made between 2007 and 2012 were selected retrospectively. All patients were treated at Erasmus MC-Sophia Children’s Hospital.

### CF patients

Inclusion criteria: diagnosis of CF; age between age 6 and 16 years; spirometer-guided chest CT acquired with SOMATOM® Definition Flash CT scanner (Siemens Healthcare, Forchheim, Germany); slow vital capacity during CT for inspiratory CT ≥85% and expiratory CT ≥80% as recommended and described by Salamon et al. [25]. Exclusion criteria: poor image quality due to motion artifacts; poor breath- hold performance as judged by a lung function technician. Twelve CF patients were randomly selected out of all patients that met the selection criteria.

### Control patients

Inclusion criteria: good or excellent spirometer-guided chest CT acquired with SOMATOM® Definition Flash scanner; clinical reason for CT other than CF; report by Erasmus MC radiologist stating chest CT to be normal; defined normal on second reading by an independent radiologist (CY, 20 years of experience in thoracic imaging) blinded to patient identifiers and information. Out of 16 control chest CTs that met the above-mentioned criteria, 12 CTs were selected with best matched ages of the CF group. More detailed control group characteristics are provided in Table 1.

**Table 1:** Diagnosis of control subjects

Clinical diagnosis	Reason for CT	Findings	No. of subjects
Asthma	Air trapping, bronchiectasis, malacia?	No air trapping, no bronchiectasis, no malacia	8
Recurrent respiratory infections	Air trapping, bronchiectasis, malacia?	No air trapping, no bronchiectasis, no malacia	3
Condition after oesophageal atresia	Tracheomalacia?	No tracheomalacia	1

## CT scanning

End-inspiratory and end-expiratory volumetric chest CTs were obtained in the supine position. Details of scan parameters are provided in Table 2.

**Table 2:** Scan parameters used to obtain the CTs

	CF subjects		Control subjects	
Scan acquisition	Inspiration	Expiration	Inspiration	Expiration
Tube voltage (kV)	80	80	(80–120)	(80–120)
Pitch	0.85	0.85	0.85	0.85
Slice thickness (mm), median (range)	1 (0.75–1)	1 (0.75–1)	1	1
Reconstruction increment (mm), median (range)	0.6 (0.3–1)	0.6 (0.3–1)	0.8 (0.6–0.8)	0.8 (0.6–0.8)
Reconstruction kernel	B70f;B75f	B70f;B75f	B75f; I70f	B75f;I50f
Collimation	128x0.6	128x0.6	128x0.6	128x0.6
Current-time product (mAs), median (range)	46 (36–52)	44 (35–53)	28 (11–61)	23 (8–65)
CTDI_vol,32 cm_ (mGy), median (range)	0.74 (0.57–0.83)	0.71 (0.56–0.84)	0.76 (0.32–1.13)	0.56 (0.24–1.05)
DLP(mGy * cm), median (range)	21 (14–28)	17 (11–25)	22.5 (7–38)	14.5 (5–30)

## Quantitative analysis of airways and arteries

**Fig. 1:**
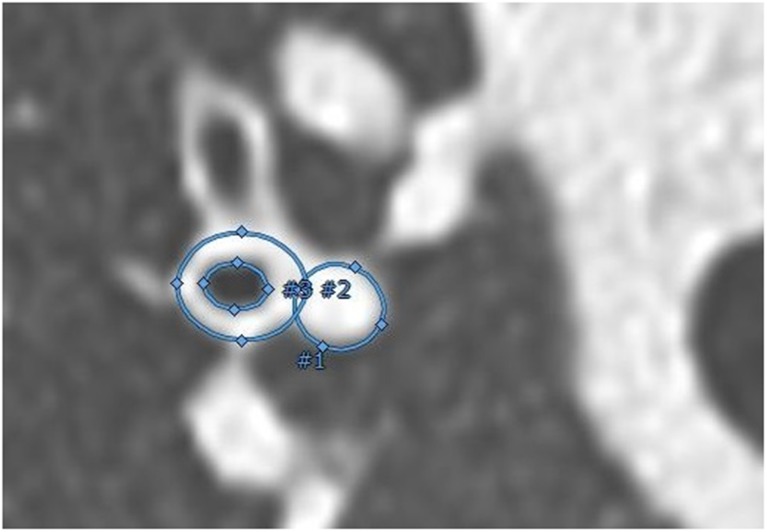
The three measurements of the airway and accompanying artery in a perpendicular view to the pathway centre-line, using an ellipse image analysis tool (Myrian®). The annotated surface areas of the inner (#3) and outer (#2) airway and of the artery (#1) were used to estimate diameters and to calculate the AA-ratios. The location of all AA-pairs was determined by using 3D segmentation (videos of rotating 3D segmentations in colour can be found in Online Supplementary Material E2)

All CTs were scored in random order using the CF-CT scoring system to quantify structural CT abnormalities in CF and controls as described in more detail separately [26]. Dimensions of AA-pairs were measured using Myrian® (v1.16.2, Lung XP module) image analysis platform (Intrasense, Montpelier, France) as described previously [26]. In summary, airway pathways were indicated automatically. Pathways of additional lobar, segmental and subsegmental airways not automatically indicated were added manually. The bronchial tree was reconstructed in a 3D-view (Online Supplementary Material, video E1), and cross-sectional CT reconstructions were generated based on the airway’s centre-line (Fig. 1). One measurement per branch was made when both airways and artery were clearly visible. AA-pairs with movement artefacts or too much noise for reliable measurements were excluded. In addition, AA-pairs of airways that did not show a visible inner lumen (e.g. due to mucous plugging) and airways without a clear identifiable adjacent artery (e.g. due to atelectasis or severe cystic bronchiectasis without a traceable artery) were excluded.

Inner and outer airway diameters were divided by artery diameter to compute A_in_A and A_out_A-ratio, respectively. Wall thickness (difference between outer and inner airway diameter) was divided by outer airway diameter to compute A_WT_-ratio and divided by artery diameter to compute A_WT_A-ratio.

### Location of airway artery measures

**Fig. 2:**
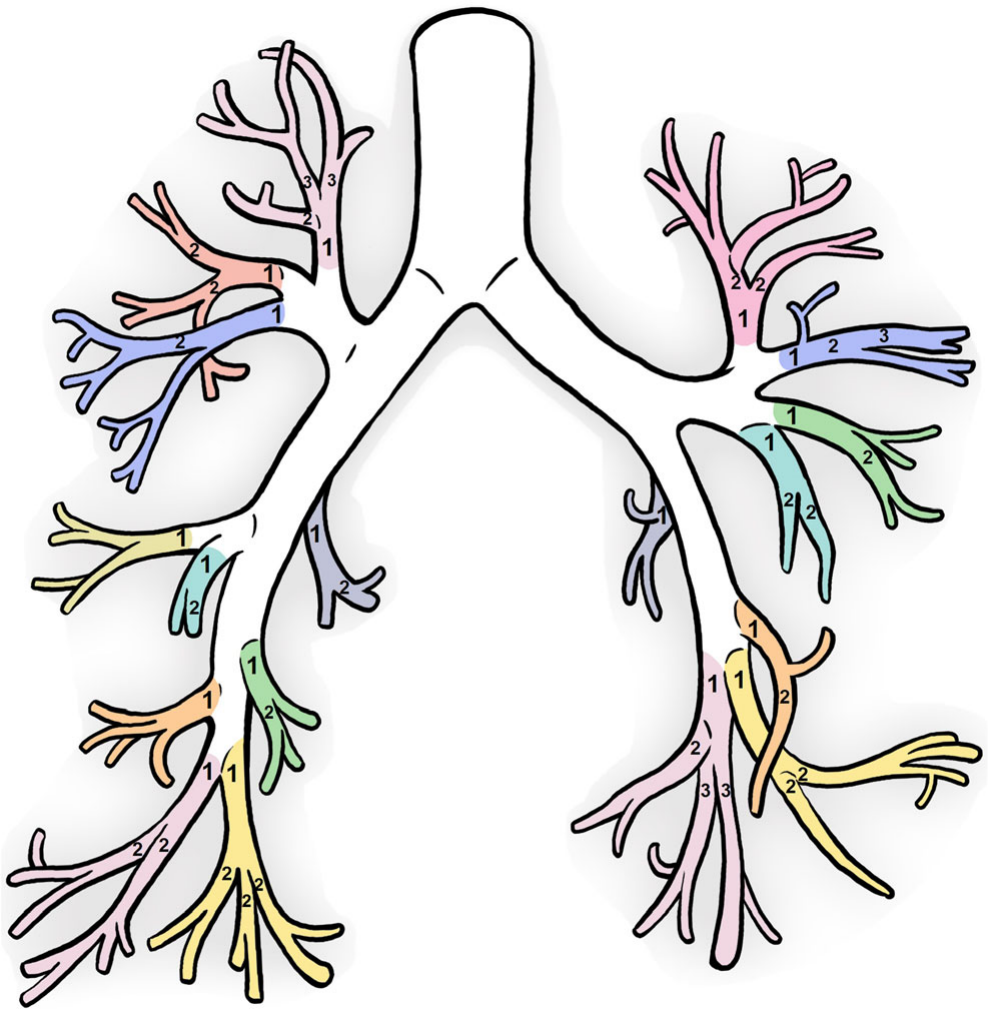
The segmental branches are shown in the different colours and the numbering stands for each segmental airway generation. The segmental generation ensured all segmental bronchi started at segmental generation 1 to avoid discrepancies in the upper and lower segmental airway generations as demonstrated in Online Supplementary Material Fig. E2 (Artist: K. Rubenis)

The lung lobes (right upper (RUL), right middle (RML), right lower (RLL), left upper (LUL), left lower lobe (LLL) and lingula (LING)), segmental bronchi (nomenclature as depicted by Netter [27]), and airway generations were annotated for each AA measurement. Airway generation started at the trachea as 0, the main stem bronchi as 1 and continuing after each time the bronchi bifurcates. The upper segmental bronchi begins at generation 3–4 and lower segmental bronchi begins at generation 4–7. The generation from each segmental bronchi as described in Online Supplementary Material Fig. E2a was subtracted from the airway generation to compute the segmental generation starting at 1 (see Fig. 2). Segmental generations ≥4 were defined as peripheral airways for the purpose of this paper.

## Observer reliability

CTs of all patients were anonymized and randomized before scoring. The scorers were blinded to all clinical information. All AA measurements were conducted by the first observer (WK, 3 years of experience). After 3 months, a total of 386 measurements were repeated in one randomly selected segmental branch of every patient to establish intra-observer variability. A second observer measured a total of 2945 AA dimensions in a random subset of 25 CTs (HO, 1 year of experience) in order to determine interobserver variability.

## Statistical analysis

Differences between patient characteristics in the two cohorts were assessed with the Mann–Whitney U test. Differences in number of AA-pairs visible were assessed using the Wilcoxon signed-rank test. The relationship between number of AA-pairs visible with height and age was evaluated with linear regression analysis. Mixed-effect models were used to predict AA-ratios using disease status and lung volume. Influence of other covariates (i.e. gender, age and height) on AA-ratios was assessed univariately. Univariate covariates found to be significant were added with interaction to the main model. Variable selection in the main model was performed with a likelihood ratio test. Mixed-effect models were used to evaluate differences between CF and control patients and volume level predicting AA-ratios for each segmental generation and lung lobes. Random-effects of lobes and individuals were included in all mixed-effect models to capture heterogeneity.

For diagnosis of bronchiectasis receiver operating characteristic (ROC) curves with the corresponding areas the under curve (AUCs) and 95% confidence interval (CI) were plotted to identify the threshold values for A_in_A and A_out_A-ratios with the highest combined sensitivity and specificity. The intraclass correlation coefficient (ICC) was used to measure inter- and intra-observer agreement, with the use of mixed-effects models. In these mixed-effects models the structure was taken into account, specifically the segmental branches within generation within patients. ICC values between 0.4 and 0.6, 0.6 and 0.8, or ≥ 0.8 were considered to indicate moderate, good, and very good agreement, respectively [28].

Results were expressed as median and interquartile range (IQR). A p-value of 0.05 or less was considered significant. All analyses were conducted using R version 3.1.0 [29].

## Results

### Study population

A detailed description of the group characteristics is shown in Table 3. One subject in the CF group was excluded after image analysis since the patient appeared to be mislabeled and diagnosed with common variable immunodeficiency. Hence, 11 patients with CF (six males) were included for further analysis with a median age of 11 years. Twelve control patients with normal CTs (seven males) were included with a median age 13.9 years. All but one control patient had spirometry performed on the same day or maximally 1 month apart from the CT scan. One control patient did not have a recent spirometry available, so spirometry of 8 months prior to the CT was used. Re-analysis without this subject did not influence differences between the groups in demographics or spirometry. No significant difference was found in age, gender, height, weight and PFTs between the two groups. All CF-CT scores were significantly higher in the CF compared to the control group.

**Table 3:** Demographics of the cystic fibrosis (CF) and normal cohorts

	Patients with CF, median (IQR)	Control patients, median (IQR)	P-value
Age at CT (years)	11 (9.3–11.1)	13.9 (8.7–15)	0.385
Age at force (years)	11 (9.3–13)	13.9 (8.7–15)	0.385
Time between CT and PFT (months)	0.0 (0.0–0.0)	0.5 (0.1–0.7)	0.011*
Gender	6 males; 5 females	7 males; 5 females	0.808
Height (cm)	144.4 (138.2–146.8)	149 (136.6–170.9)	0.296
Weight (kg)	34.5 (30.4–45.3)	40.1 (28.6–65.8)	0.461
BMI	17.5 (15.5–19.4)	18.1 (15.9–20.2)	0.435
CF-CT BE score (%)	5.2 (1.4–12.1)	0.0 (0.0–0.7)	<0.001*
CF-CT AWT score (%)	5.6 (0.0–19.3)	0.0 (0.0–0.0)	<0.001*
CF-CT MP score (%)	2.8 (0.0–22.9)	0.0 (0.0–0.0)	<0.001*
CF-CT AT score (%)	51.2 (25.9–66.7)	3.7 (0.0–8.3)	<0.001*
CF-CT total score (%)	7.8 (6.2–18.4)	1.2 (0.4–1.4)	<0.001*
FEV_1_ (z-scores)	− -1.3 (− -2.2–0.1)	− -1.7 (− -2.1– -0.4)	0.668
FVC (z-scores)	− -0.1 (− -1.2–0.8)	− -0.4 (− -2.3–0.8)	0.409
FEV_1_/FVC	0.82 (0.76–0.83)	0.82 (0.70–0.91)	0.385
FEF_25–75_ (z-scores)	− -1.8 (− -2.3– -0.6)	− -1.4 (− -2.5– -1)	0.939

Wilcoxon signed rank test to test the difference in demographics between the CF and control group. CF-CT scores of mucous plugging (MP), air trapping (AT), and the total score were compared as well as CF-CT scores of BE and AWT. Spirometry was compared using Z-scores according to Quanjer et al. [41]

*PFT* pulmonary function test, *BMI* body mass index, *FEV*_*1*_ forced expiratory flow in 1 s, *FVC* forced vital capacity, *FEF*_*25-75*_ forced expiratory flow during the 25-75% portion of the FVC

#### Quantitative analysis of airways and arteries

**Fig. 3:**
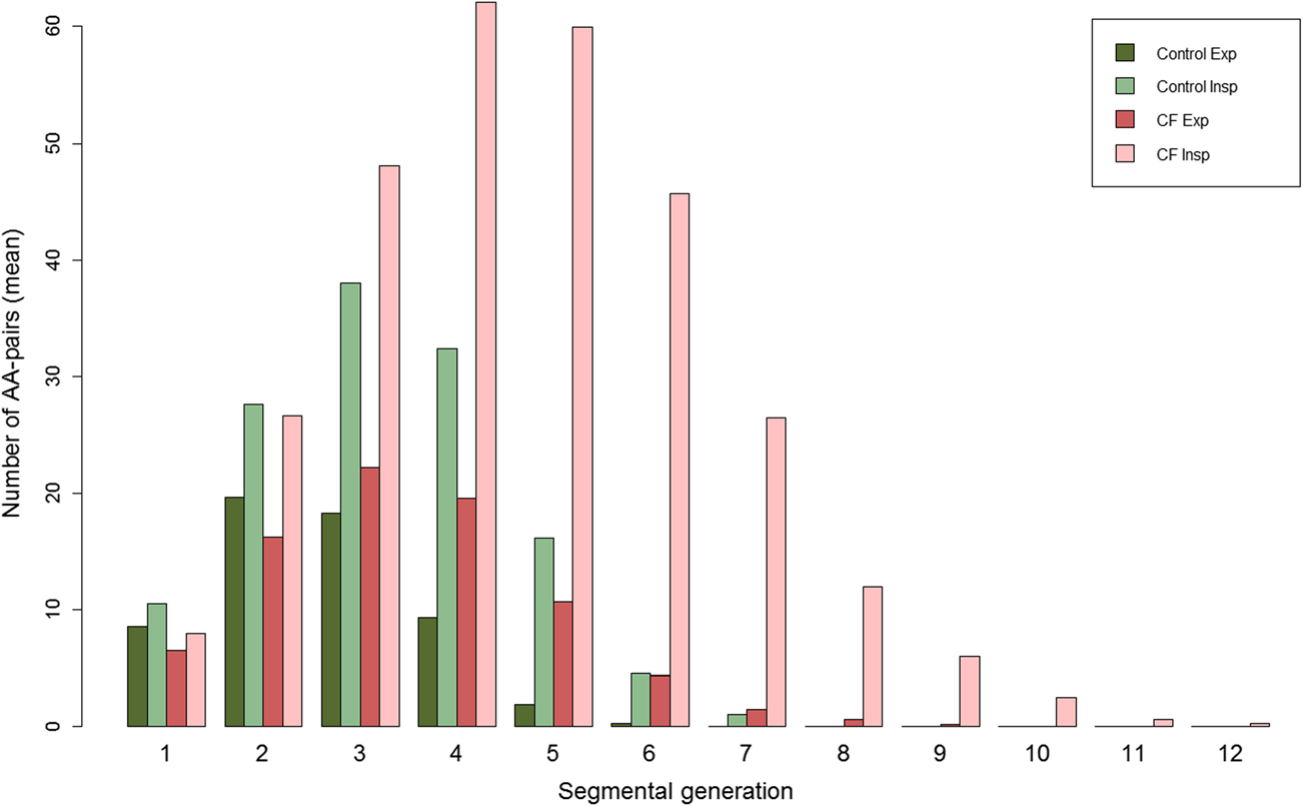
Average number of AA-pairs visible per segmental generation for controls (green) and cystic fibrosis (CF) patients (red), separated by inspiratory (light colours) and expiratory (dark colours) CT. Note that the total number of visible airways on the inspiratory scans in CF is increased relative to controls

A total of 6,464 AA-pairs were measured in perpendicular view of the airway axis. In CF patients a mean of 299 AA-pairs were measured on end-inspiration and 82 AA-pairs on the end-expiration CTs. In control patients a mean of 131 pairs were measured on end-inspiration and 58 AA-pairs on the end-expiration CTs. Number of visible AA-pairs was significantly higher in inspiration compared to expiration CTs in both CF and control groups (p<0.001). This difference is especially prominent after segmental generation 4 (Fig. 3). CF patients had significantly more visible AA-pairs (p<0.001) than controls in inspiratory CTs, but not in expiratory CTs (p=0.54). As seen in Fig. 3, patients with CF have a large number of visible AA-pairs in higher generations (8–12), whereas control patients had none. Number of AA-pairs counted was not correlated with age (p=0.72) or height (p=0.77).

#### Airway-artery dimensions

**Fig. 4:**
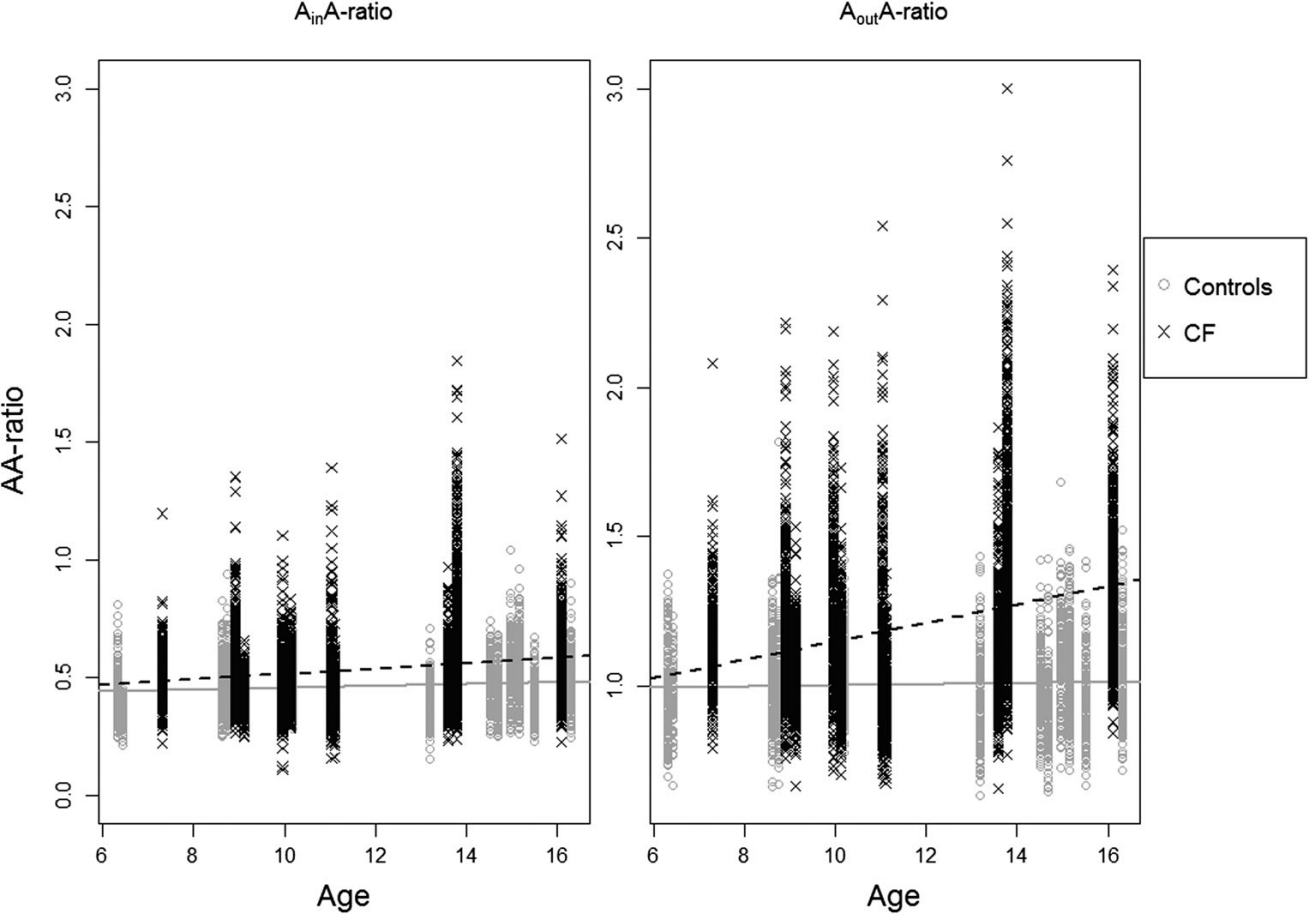
The AA-ratio is plotted as a function of age, for A_in_A (left) and A_out_A (right). The cystic fibrosis (CF) patients are depicted as black X and dashed line and the control patients as grey O and solid lines. A_in_A-ratio remained constant with increasing age in the CF (value=0.010, p=0.28) and control (value=0.006, p=0.22) groups. The A_out_A-ratio in the CF group (value=0.026, p=0.10) and control group (value=0.002, p=0.52) also did not increase significantly with age

Median (range) of the inner, outer, wall and vessel diameter were 1.65 (0.36–6.58), 3.74 (1.00–9.84), 2.08 (0.50–6.28) and 3.42 (0.94–11.99) mm, respectively (Online Supplementary Material Fig. E3). A_in_A- and A_out_A-ratio was independent of age in both CF and control patients (Fig. 4). Gender, age and height were not significantly related to A_in_A, A_out_A or A_WT_A-ratio in a univariate mixed-effect model. Height, but not gender and age, was found to be significant in the mixed-effect model with A_WT_-ratio (p=0.001), so height was included in the model describing A_WT_-ratio.

A_in_A, A_out_A, A_WT_ and A_WT_A-ratios differed significantly on inspiratory CTs compared to expiratory CTs for both CF and control groups (P<0.001). The significant differences in AA- and AWT-ratios between CF and control patients are shown in Table 4.

**Table 4:** Comparison of airway dimensions between cystic fibrosis (CF) patients and controls

Significant difference between disease status (Control/CF)	A_in_A-ratio	A_out_A-ratio	A_WT_A-ratio	A_WT_-ratio
Inspiration	0.156	<0.001*	<0.001*	0.215
Expiration	0.996	0.003*	<0.001*	0.036*

P-values of mixed-effect model analysis of differences between airway dimensions between CF and control patients. A_out_A and A_WT_A -ratios were higher in CF patients than controls in both inspiratory and expiratory scans. A_in_A-ratios was not significant and the A_WT_-ratio only in the expiratory subgroup

#### Comparison of CF versus control group

The optimal threshold to define BE was reached at a value of 0.5 for A_in_A-ratio and 1.11 for A_out_A-ratio. AUC (95% CI) for A_in_A was 0.6 (0.59–0.61) and 0.72 (0.71–0.74) for A_out_A-ratio (Online Supplementary Material Fig. E4). The optimal threshold for BE of peripheral airways (segmental generation ≥4) was 0.56 for A_in_A-ratio (AUC 0.63, 95% CI 0.61–0.65) and 1.17 for A_out_A-ratio (AUC 0.75, 95% CI 0.73–0.77).

#### Location of airway and artery measures

**Fig. 5:**
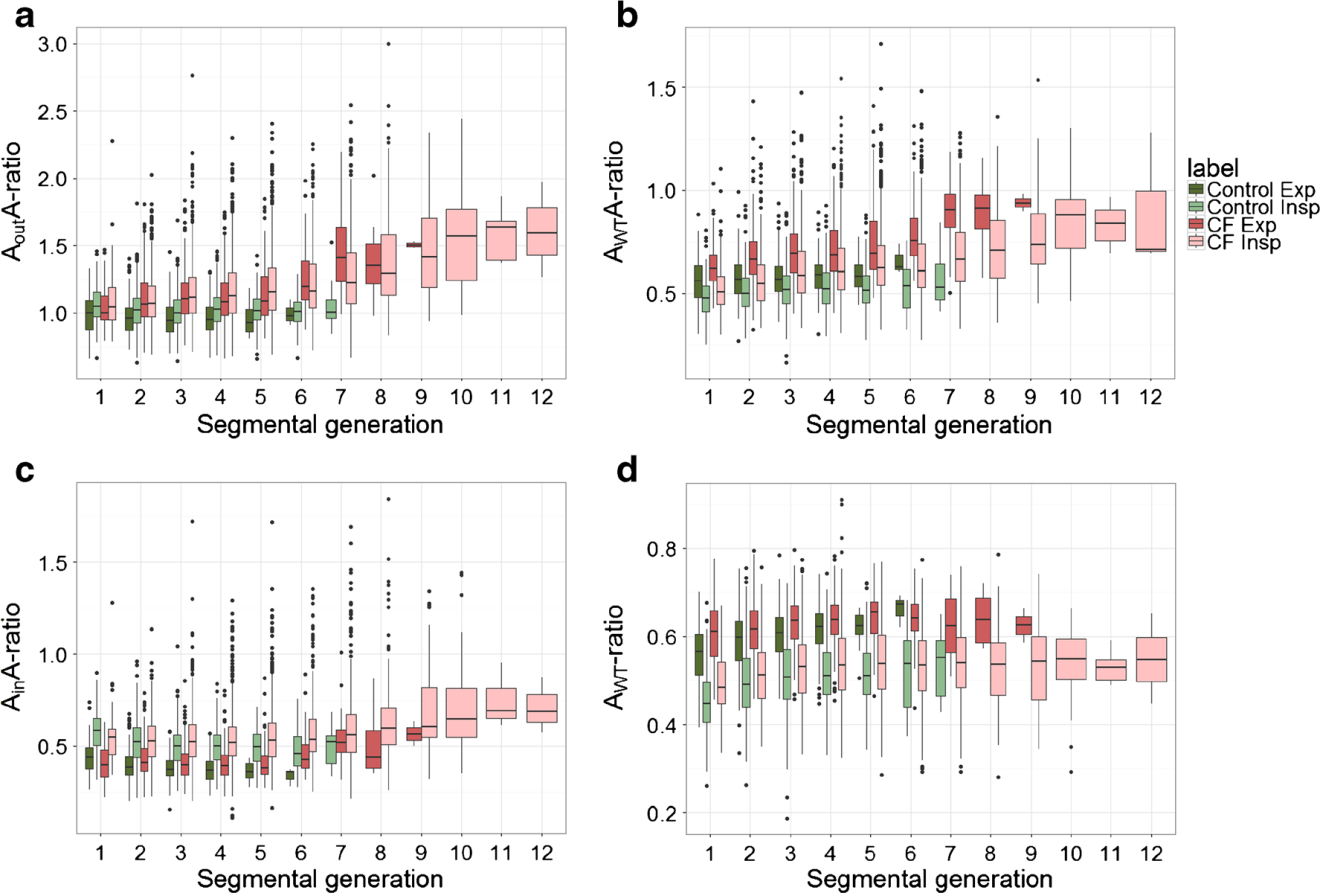
Boxplots of the AA-ratios in the control group (green) and in the cystic fibrosis (CF) group (red) for each segmental generation. Inspiratory scans are shown in light colours and expiratory scans in dark colours. Each box shows median (horizontal line), interquartile range (solid box), 1.5*interquartile range (whiskers) and outliers (points). **a** When focusing on the inspiratory scans, an increasing difference can be found in A_out_A-ratio between the CF and control group from generation 2 to generation 6 (increasing difference of 0.08–0.19, p≤0.02). The difference between CF and the control group of the A_out_A-ratio in the expiratory scans was found to be significant from generation 2 to 5 (difference of 0.13–0.15, p≤0.04). **b** On the inspiratory scans, a difference can be found in A_WT_A-ratio between the cystic fibrosis (CF) and control groups starting from segmental generation 1 to 6 (difference of 0.05–0.12, p≤0.04). The difference in A_WT_A-ratio between CF and the control group in the expiratory scans was found to be significant from generation 1 to 5 (difference of 0.08–0.13, p≤0.003). **c** There was no difference in A_in_A-ratio between the cystic fibrosis (CF) and control groups on inspiratory CTs (p≥0.08), neither was there for the expiratory scans (p≥0.28). A difference in A_in_A-ratio was observed between inspiration and expiration CTs of both the CF (difference of 0.12–0.20, p≤0.04) and control group (difference of 0.13–0.14, p≤0.001). **d** There was no difference in A_WT_-ratio between the cystic fibrosis (CF) and control groups in all segmental generations on the inspiratory CTs. On expiratory CTs only a difference was found in segmental generation 1 (difference of 0.05, p=0.021). An apparent difference was found between inspiratory and expiratory scans in A_WT_-ratio for the CF group (0.08–0.11, p≤0.001), as well for the control group (0.09–0.15, p≤0.001) in all segmental generations (all absolute values can be found in Online Supplemental Material Table E1a–d)

Figure 5a and b show A_out_A and A_WT_A-ratio by segmental generation. The difference in A_out_A and A_WT_A-ratio between inspiratory CF and controls was significant for segmental generation 2–6 (p≤0.02). Difference in A_out_A between patients with CF and control patients became larger with each following segmental generation. A_in_A and A_WT_-ratio did not differentiate significantly between the CF and control group on inspiratory CT in each segmental generation (Fig. 5c, d). More detail on the differences and significance values for each segmental generation can be found in Online Supplemental Material Table E1a-d.

Quantitatively, bronchiectasis has an upper lobe predominance. Online Supplemental Material Table E2a-d shows the detailed results of the mixed-effect model regarding the different lung lobes. In both CF and control patients, A_out_A-, A_WT_A- and A_WT_-ratio were significantly higher in RUL compared to all other lobes (p<0.001). In CF patients, A_out_A- and A_WT_A-ratio was significantly higher in LUL compared to right middle lobe (RML), right lower lobe (RLL) and left lower lobe (LLL) (p<0.001). LLL was significant lower than all other lobes for A_out_A-, A_WT_A-, and A_WT_-ratio in CF patients (p≤0.004).

#### Reproducibility of airway measurements

For the interobserver variability, ICC for AA dimensions were as follows: Inner airway (0.69), outer airway (0.72), wall (0.66) and vessel diameter (0.69). For the intra-observer variability, ICC for the different dimensions were as follows: Inner airway (0.70), outer airway (0.74), wall (0.71) and vessel diameter (0.79).

## Discussion

To our knowledge, this is the first study measuring all visible AA-pairs perpendicular to airway centre-lines in maximal inspiration and expiration CTs of CF patients and control patients. Major differences were observed in number of visible AA-pairs and in AA-ratios between inspiratory and expiratory CTs and between CF patients and control patients.

Our study showed that end-inspiratory CTs were most sensitive to detect structural airway disease. Overall two to three times more AA-pairs were detected on end-inspiratory CTs of a patient with CF than on CTs of controls. Difference in number of visible AA-pairs became more striking with each generation. Beyond the seventh segmental generation, AA-pairs were still visible in CF but not in control patients. Our study confirms that number visible of AA-pairs could be used as a surrogate outcome for bronchiectasis [26, 30–32]. Nevertheless, it is important to keep in mind that the number of visible airways can be affected by patient size as well as inspiration level and scan protocol. In this study, patient size did not influence the number of AA-pairs counted and scan protocol did not play a role as inspiratory CTs of both CF and control patients were scanned using the same scanner and protocols.

A_out_A and A_WT_A-ratios on inspiratory CTs progressively increased with segmental generation in CF subjects, while they remained relatively constant over generations in control subjects. Increased AA-ratios and higher number of visible peripheral airways suggests more structural abnormalities due to CF lung disease in the more peripheral airways compared to central airways. These are findings that to our knowledge have not been established by quantitative assessment previously and strongly support the importance of peripheral airways in CF lung disease [32–35]. An increase in visible AA-pairs or in AA-ratios might, however, easily be missed on visual routine CT.

AA-ratio based on outer airway diameter was more sensitive to detect BE compared to inner diameter as demonstrated by the higher AUC in the ROC curve. Furthermore, the A_out_A-ratio was less influenced by the inspiration level compared to the A_in_A-ratio. Lastly, A_in_A-ratio could not differentiate between CF and controls within all segmental generations. This can be explained by simultaneous airway wall thickening due to inflammation and mucus impaction, leading to decreased A_in_A-ratio. This finding supports previous [11] and recently published guidelines [12, 26] that rely on outer airway diameter for diagnosis of bronchiectasis on CT. A lower AA-ratio was reported in children by Kapur et al. [5], but this was based on the inner luminal diameter instead of the outer diameter. In our dataset, an AA-ratio based on an outer diameter of 1.1 was optimal to differentiate between normal and abnormal. However, A_out_A-ratios above 1.1 were found in the control group as well.

Number of AA-pairs visible and AA-ratios between the groups were only significantly different in end-inspiration and not in end-expiration CT. Additionally AWT values were higher in end-expiratory CTs, possibly due to folding of the airways in expiration [33]. Thus, in case an ‘inspiratory’ CT is performed below TLC, number of bronchiectatic airways will be underestimated. On the other hand AWT increased and became more apparent in end-expiratory. Hence, when lung volume is below TLC during CT acquisition, AWT could be overestimated. These findings strongly support the need for volume control during acquisition to allow consistent, sensitive and objective detection of BE and AWT [7, 25].

In control patients the AA-ratio did not change significantly with age, as also reported in previous studies [5, 21]. In CF a trend was observed of an increasing A_out_A-ratio with age, which can be due to disease progression. Nevertheless, we dealt with a small sample size, thus a larger study population is needed to investigate changes in the AA-ratios according to age.

Higher A_out_A-ratios were observed in the upper lobes, especially the RUL. This observation is in in accord with previous publications observing more severe abnormalities and inflammation in the RUL of CF patients [13, 36, 37]. Hence, bronchiectasis detection most likely has to take lobes into account. Intra-subject differences in airway dimensions between lung lobes have previously been reported in COPD patients [23, 24, 38] and control subjects [22].

Our study has several limitations. First, the control patients were not healthy as they were referred for chest CT through the paediatric respiratory department. Even though all CTs were reported to be normal by two independent radiologists, we cannot exclude that subtle changes in AA dimensions could have influenced our comparisons. Secondly, scan and reconstruction parameters were not identical in all CTs of this retrospective study. In theory this could have caused a small bias, but we believe this bias to be small at most since there was no automated post-processing involved in detecting the airway and artery dimensions, but instead they were measured manually with an ellipse tool. However, it is unlikely that these limitations can explain the large differences found between CF and control patients. Importantly, a constant AA-ratio was observed for each airway generation in control patients while this ratio steadily increased in CF. Moreover, the CF-CT scored almost no abnormalities in control subjects, except for some trapped air which can be physiological in healthy patients [39]. A second limitation is that we only studied a relatively small population. This was due to the time needed to manually score all 48 inspiratory and expiratory CTs (average of 15 h per scan). However, a large number of AA-pairs could be measured to test our research questions. Thirdly, radiation dose for expiratory CTs was lowered after 2012 in five out of 12 control patients. This reduced the sensitivity of measurements and could have reduced the number of visible AA-pairs, leading to overestimation of the effect of inspiration level in control patients. Finally, reproducibility of single AA measurements was hard to assess as repeated manual measurements were unlikely to be measured at identical anatomical locations, causing variability. However ICCs showed good inter- and intra-observer agreements.

In conclusion, the results of this study showed greater structural lung changes with each airway generation on chest CTs of children with CF. In children with CF, more airways were visible on CT due to the increase of AA-ratios in the peripheral part of the lungs. For diagnosis and quantification of BE outer airway diameter should be used, as this is a better differentiator between CF and control patients. Our findings strongly support the need for volume standardization in chest CTs of children aged 6 years and above, for objective and sensitive evaluation of airway dimensions [7]. To generate reference values for airway dimensions in children, a larger study population of normal chest CTs acquired at TLC is needed. Automatic methods to quantify bronchiectasis are in development to support clinicians’ diagnosis and reduce the time of annotations [40].

## Electronic supplementary material

Below is the link to the electronic supplementary material.

ESM 1 (DOCX 797 kb)
