# Supplementary material for: Diagnosis of bronchiectasis and airway wall thickening in children with cystic fibrosis: Objective airway-artery quantification
Source: Eur Radiol. 2017 May 18;27(11):4680–9. doi: 10.1007/s00330-017-4819-7 (PMC5635089; doi:10.1007/s00330-017-4819-7)
Supplement: Supplementary file 1 — (DOCX 797 kb) [file 330_2017_4819_MOESM1_ESM.docx]

**Diagnosis of bronchiectasis in children with cystic fibrosis: Objective airway-artery quantification**

Wieying Kuo^1, 2^, Marleen de Bruijne, PhD^3,4^, Jens Petersen, PhD^4^, Kazem Nasserinejad^5^, Hadiye Ozturk^1^, Yong Chen^6^, MD, Adria Perez-Rovira, PhD^1,3^, Harm A.W.M. Tiddens, MD, PhD^1,2^

Online Data Supplement

**MATERIAL AND METHODS**

**Online supplement E1**

*CF subjects*: Patients with CF were monitored routinely with a biennial chest CT. Since 2007, a protocol for spirometer guided inspiratory and expiratory chest CT was introduced for all chest CTs to optimize and standardize lung volume and reduce movement artefacts. Consequently all CTs of CF patients were spirometer guided (1).

*Disease control subjects:* Indication for CTs in this control group can be found in **table 1**.

Pulmonary function test
Routine pulmonary function tests (PFT) were performed in all CF patients and controls. For both groups PFT closest to, or on the same day, of CT acquisition was used for analysis. The following parameters were included and expressed as Z-scores: FEV_1_, FVC, forced expiratory flows between 25 and 75% of expiratory VC (FEF_25-75_) (2). Age at the day of the CT was used to define patient characteristics. For height and weight we used values as recorded at the time of PFT.

**Tables**

**Table Online supplement E1a**The following tables describe the mean difference and p-value as depicted by the mixed-effect model for A_out_A-ratio in each generation as plotted in the figure 4a.

| **A_out_A-ratio** | 1 | 2 | 3 | 4 | 5 | 6 | 7 | 8 | 9 |
| --- | --- | --- | --- | --- | --- | --- | --- | --- | --- |
| Control insp vs CF insp | (p=0.53) | 0.08 (p=0.019) | 0.13 (p<0.001) | 0.13 (p<0.001) | 0.17 (p<0.001) | 0.19 (p=0.004) | (p=0.15) |  |  |
| Control insp vs control exp | -0.08 (p<0.001) | -0.07 (p<0.001) | -0.08 (p<0.001) | -0.08 (p<0.001) | (p=0.15) | (p=0.89) |  |  |  |
| Control insp vs CF exp | (p=0.71) | 0.08 (p=0.022) | (p=0.09) | (p=0.21) | (p=0.17) | (p=0.06) | (p=0.34) |  |  |
| Control exp vs CF exp | (p=0.06) | 0.15 (p<0.001) | 0.15 (p<0.001) | 0.13 (p=0.003) | 0.14 (p=0.036) | (p=0.30) |  |  |  |
| CF insp vs CF exp | (p=0.16) | (p=0.98) | -0.06 (p<0.001) | -0.08 (p<0.001) | -0.10 (p<0.001) | (p=0.13) | (p=0.54) | (p=0.50) | (p=0.59) |

**Table Online supplement E1b**The following tables describe the mean difference and p-value as depicted by the mixed-effect model for A_WT_A-ratio in each generation as plotted in the figure 4b.

| **A_WT_A-ratio** | 1 | 2 | 3 | 4 | 5 | 6 | 7 | 8 | 9 |
| --- | --- | --- | --- | --- | --- | --- | --- | --- | --- |
| Control insp vs CF insp | 0.05 (p=0.037) | 0.06 (p=0.004) | 0.09 (p<0.001) | 0.09 (p=0.001) | 0.12 (p<0.001) | 0.12 (p=0.004) | (p=0.10) |  |  |
| Control insp vs control exp | 0.06 (p<0.001) | 0.06 (p<0.001) | 0.05 (p<0.001) | 0.06 (p<0.001) | 0.07 (p=0.049) | (p=0.15) |  |  |  |
| Control insp vs CF exp | 0.15 (p<0.001) | 0.17 (p<0.001) | 0.18 (p<0.001) | 0.17 (p<0.001) | 0.20 (p<0.001) | 0.23 (p<0.001) | 0.22 (p=0.006) |  |  |
| Control exp vs CF exp | 0.08 (p=0.002) | 0.12 (p<0.001) | 0.13 (p<0.001) | 0.12 (p<0.001) | 0.13 (p=0.003) | (p=0.37) |  |  |  |
| CF insp vs CF exp | 0.10 (p<0.001) | 0.12 (p<0.001) | 0.09 (p<0.001) | 0.08 (p<0.001) | 0.08 (p<0.001) | 0.11 (p<0.001) | 0.11 (p=0.011) | (p=0.26) | (p=0.58) |

**Table Online supplement E1c**The following tables describe the mean difference and p-value as depicted by the mixed-effect model for A_in_A-ratio in each generation as plotted in the figure 4c.

| **A_in_A-ratio** | 1 | 2 | 3 | 4 | 5 | 6 | 7 | 8 | 9 |
| --- | --- | --- | --- | --- | --- | --- | --- | --- | --- |
| Control insp vs CF insp | (p=0.28) | (p=0.38) | (p=0.09) | (p=0.09) | (p=0.08) | (p=0.09) | (p=0.38) |  |  |
| Control insp vs control exp | -0.14 (p<0.001) | -0.13 (p<0.001) | -0.13 (p<0.001) | -0.13 (p<0.001) | -0.14 (p<0.001) | (p=0.08) |  |  |  |
| Control insp vs CF exp | -0.16 (p<0.001) | -0.09 (p=0.002) | -0.11 (p<0.001) | -0.12 (p<0.001) | -0.13 (p<0.001) | -0.09 (p=0.042) | (p=0.48) |  |  |
| Control exp vs CF exp | (p=0.58) | (p=0.28) | (p=0.45) | (p=0.72) | (p=0.85) | (p=0.50) |  |  |  |
| CF insp vs CF exp | -0.13 (p<0.001) | -0.12 (p<0.001) | -0.16 (p<0.001) | -0.17 (p<0.001) | -0.18 (p<0.001) | -0.16 (p<0.001) | -0.13 (p=0.012) | -0.20 (p=0.035) | (p=0.21) |

**Table Online supplement E1d**The following tables describe the mean difference and p-value as depicted by the mixed-effect model for A_WT_-ratio in each generation as plotted in the figure 4d.

| **A_WT_-ratio** | 1 | 2 | 3 | 4 | 5 | 6 | 7 | 8 | 9 |
| --- | --- | --- | --- | --- | --- | --- | --- | --- | --- |
| Control insp vs CF insp | (p=0.06) | (p=0.53) | (p=0.36) | (p=0.33) | (p=0.20) | (p=0.37) | (p=0.51) |  |  |
| Control insp vs control exp | 0.10 (p<0.001) | 0.09 (p<0.001) | 0.09 (p<0.001) | 0.10 (p<0.001) | 0.11 (p<0.001) | 0.15 (p<0.001) |  |  |  |
| Control insp vs CF exp | 0.15 (p<0.001) | 0.11 (p<0.001) | 0.13 (p<0.001) | 0.13 (p<0.001) | 0.09 (p<0.001) | 0.13 (p<0.001) | 0.10 (p=0.006) |  |  |
| Control exp vs CF exp | 0.05 (p=0.021) | (p=0.19) | (p=0.07) | (p=0.12) | (p=0.20) | (p=0.67) |  |  |  |
| CF insp vs CF exp | 0.11 (p<0.001) | 0.11 (p<0.001) | 0.11 (p<0.001) | 0.11 (p<0.001) | 0.12 (p<0.001) | 0.11 (p<0.001) | 0.08 (p<0.001) | 0.11 (p<0.001) | (p=0.11) |

**Table Online supplement E2a**The following tables describe the mean difference and p-value for A_out_A-ratio in each lobe computed with the mixed-effect model.

| **A_out_A-ratio** | RUL | | RML | | RLL | | LUL | | LING | |
| --- | --- | --- | --- | --- | --- | --- | --- | --- | --- | --- |
|  | CF | Control | CF | Control | CF | Control | CF | Control | CF | Control |
| RUL |  |  |  |  |  |  |  |  |  |  |
| RML | -0.11 (p<0.001) | -0.02 (p=0.048) |  |  |  |  |  |  |  |  |
| RLL | -0.10 (p<0.001) | -0.03 (p<0.001) | (p=0.36) | (p=0.24) |  |  |  |  |  |  |
| LUL | -0.06 (p<0.001) | -0.03 (p<0.001) | 0.05 (p<0.001) | (p=0.30) | 0.04 (p<0.001) | (p=0.97) |  |  |  |  |
| LING | -0.09 (p<0.001) | -0.03 (p=0.039) | (p=0.28) | (p=0.65) | (p=0.58) | (p=0.71) | (p=0.20) | (p=0.72) |  |  |
| LLL | -0.15 (p<0.001) | -0.04 (p<0.001) | -0.03 (p=0.02) | (p=0.13) | -0.05 (p<0.001) | (p=0.57) | -0.08 (p<0.001) | (p=0.67) | -0.06 (p=0.004) | (p=0.50) |

**Table Online supplement E2b**The following tables describe the mean difference and p-value for A_WT_A-ratio in each lobe computed with the mixed-effect model.

| **A_WT_A-ratio** | RUL | | RML | | RLL | | LUL | | LING | |
| --- | --- | --- | --- | --- | --- | --- | --- | --- | --- | --- |
|  | CF | Control | CF | Control | CF | Control | CF | Control | CF | Control |
| RUL |  |  |  |  |  |  |  |  |  |  |
| RML | -0.08 (p<0.001) | -0.02 (p=0.005) |  |  |  |  |  |  |  |  |
| RLL | -0.08 (p<0.001) | -0.03 (p<0.001) | (p=0.67) | (p=0.44) |  |  |  |  |  |  |
| LUL | -0.05 (p<0.001) | -0.02 (p<0.006) | 0.03 (p=0.006) | (p=0.77) | 0.03 (p<0.001) | (p=0.22) |  |  |  |  |
| LING | -0.07 (p<0.001) | -0.02 (p=0.024) | (p=0.56) | (p=0.94) | (p=0.37) | (p=0.62) | (p=0.16) | (p=0.75) |  |  |
| LLL | -0.12 (p<0.001) | -0.03 (p<0.001) | -0.04 (p<0.001) | (p=0.23) | -0.04 (p<0.001) | (p=0.52) | -0.07 (p<0.001) | (p=0.09) | -0.05 (p<0.001) | (p=0.39) |

**Table Online supplement E2c**The following tables describe the mean difference and p-value for A_in_A-ratio in each lobe computed with the mixed-effect model.

| **AinA-ratio** | RUL | | RML | | RLL | | LUL | | LING | |
| --- | --- | --- | --- | --- | --- | --- | --- | --- | --- | --- |
|  | CF | Control | CF | Control | CF | Control | CF | Control | CF | Control |
| RUL |  |  |  |  |  |  |  |  |  |  |
| RML | -0.3 (p<0.001) | (p=0.87) |  |  |  |  |  |  |  |  |
| RLL | -0.02 (p=0.017) | (p=0.41) | (p=0.08) | (p=0.44) |  |  |  |  |  |  |
| LUL | (p=0.16) | (p=0.07) | 0.02 (p=0.03) | (p=0.11) | (p=0.48) | (p=0.23) |  |  |  |  |
| LING | (p=0.18) | (p=0.64) | (p=0.31) | (p=0.60) | (p=0.90) | (p=0.99) | (p=0.61) | (p=0.46) |  |  |
| LLL | -0.02 (p=0.002) | (p=0.39) | (p=0.29) | (p=0.42) | (p=0.37) | (p=0.93) | (p=0.14) | (p=0.28) | (p=0.73) | (p=0.97) |

**Table Online supplement E2d**The following tables describe the mean difference and p-value for A_WT_-ratio in each lobe computed with the mixed-effect model.

| **A_WT_-ratio** | RUL | | RML | | RLL | | LUL | | LING | |
| --- | --- | --- | --- | --- | --- | --- | --- | --- | --- | --- |
|  | CF | Control | CF | Control | CF | Control | CF | Control | CF | Control |
| RUL |  |  |  |  |  |  |  |  |  |  |
| RML | -0.01 (p=0.005) | (p=0.11) |  |  |  |  |  |  |  |  |
| RLL | -0.02 (p<0.001) | -0.01 (p=0.049) | (p=0.22) | (p=0.82) |  |  |  |  |  |  |
| LUL | -0.01 (p=0.007) | (p=0.83) | (p=0.54) | (p=0.20) | 0.01 (p=0.018) | (p=0.16) |  |  |  |  |
| LING | -0.02 (p=0.013) | (p=0.33) | (p=0.68) | (p=0.80) | (p=0.68) | (p=0.91) | (p=0.37) | (p=0.44) |  |  |
| LLL | -0.03 (p<0.001) | -0.01 (p=0.024) | -0.02 (p<0.001) | (p=0.91) | -0.02 (p<0.001) | (p=0.64) | -0.03 (p<0.001) | (p=0.09) | -0.02 (p=0.008) | (p=0.71) |

**Figure legends**

**Figure Online supplement E1**

Video of rotating 3D segmentations

**Figure Online supplement E2**


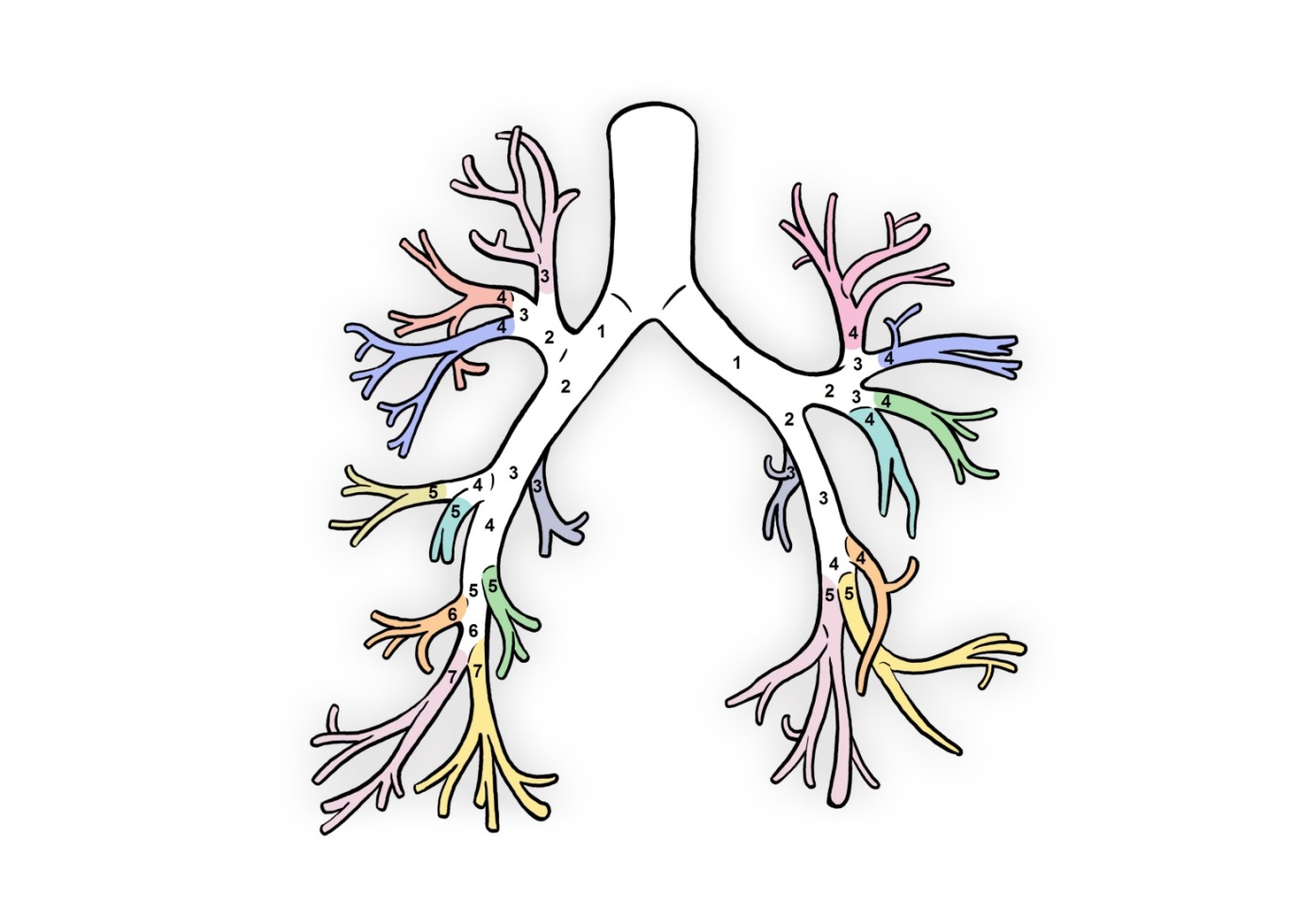


Figure Online supplement E2. Anatomy of the airways. The segmental branches are shown in the different colors and the numbering stands for the airway generation. Each segmental branch starts with a different generation, eg generation 3-4 in the upper segmental branches whereas it starts at generation 4-7 in the lower segmental branches (Artist: K. Rubenis).

**Figure Online supplement E3
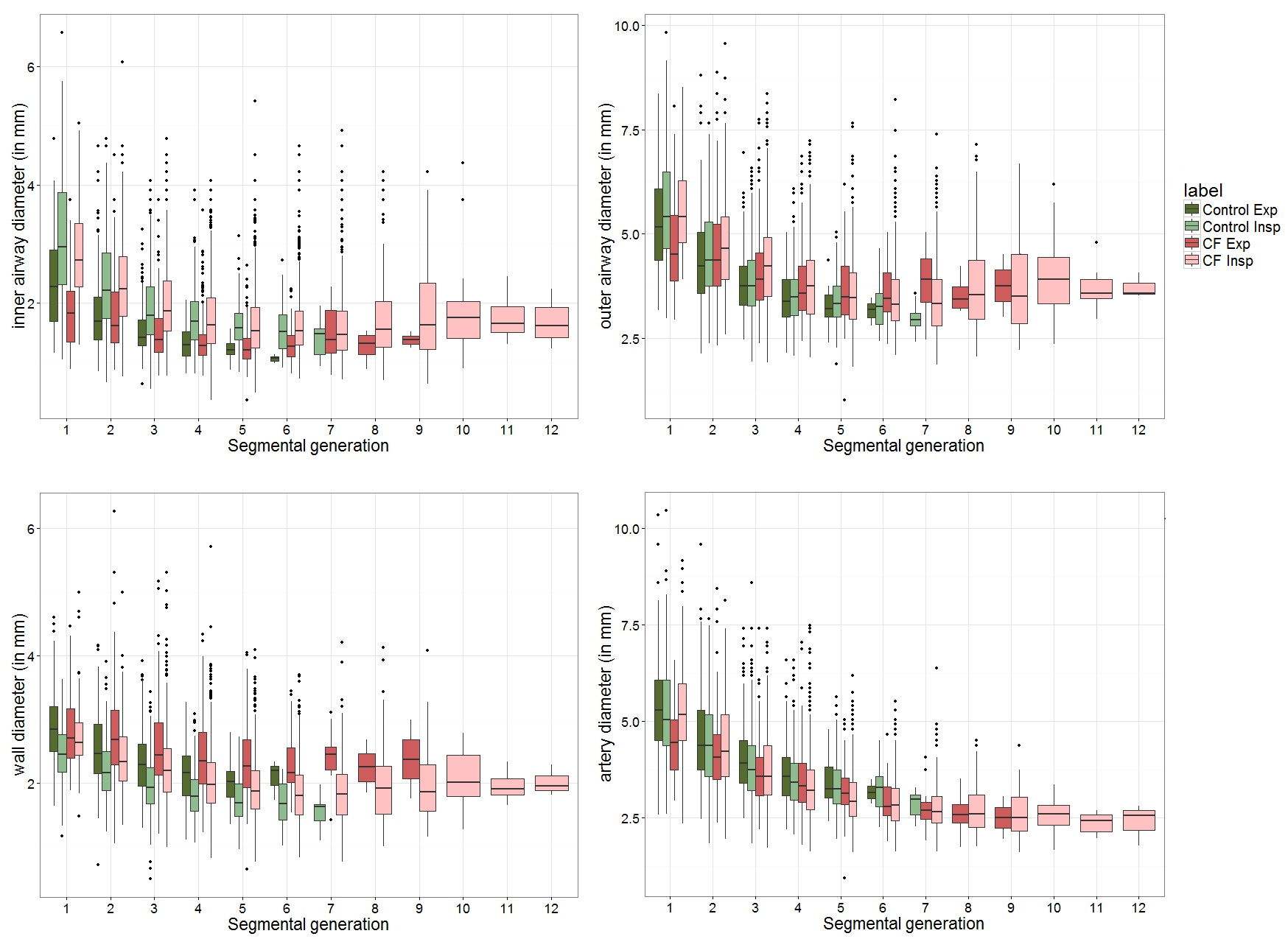
**Figure Online supplement E3. Boxplots of the inner and outer airway diameter (top graphs) and the airway wall and artery diameter (bottom graphs. The diameters are shown for the control group (green) and the CF group (red) for each segmental generation. Inspiratory scans are shown in light colors and expiratory scans in dark colors. Each box shows median (horizontal line), interquartile range (solid box), 1.5*interquartile range (whiskers) and outliers (points).

**Figure Online supplement E4**


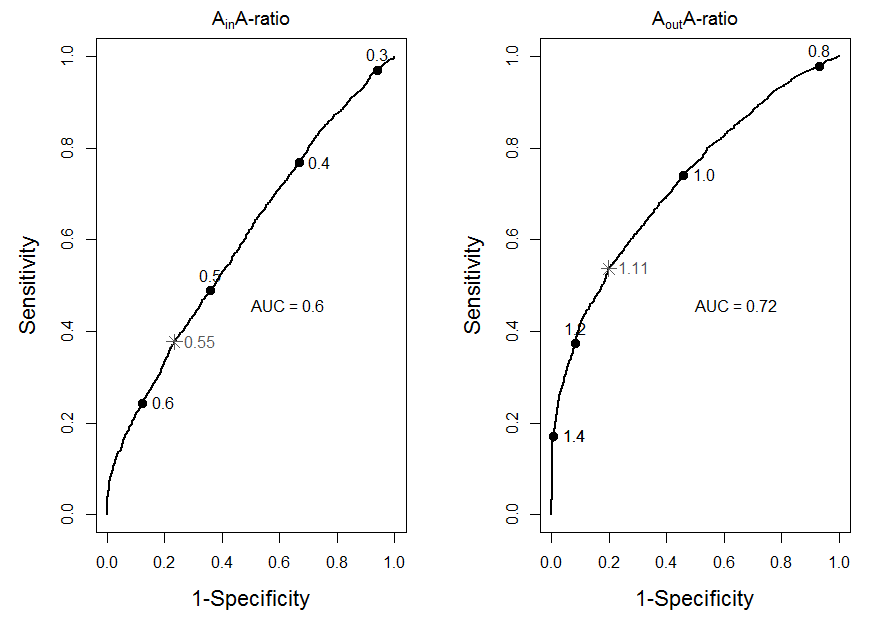


Figure Online supplement E4. ROC curves to distinguish between patients with CF and control subjects show that A_out_A-ratio (right) had better detection capability than A_in_A-ratio (left). The gray stars indicate the AA-ratio with optimal sensitivity + specificity.

**References**

1. Salamon ER, Lever S, Kuo W, Ciet P, Tiddens HAWM. Spirometer guided chest imaging in children: It is worth the effort. Pediatr Pulmonol. 2015;in Press(April):1–9.

2. Quanjer PH, Stanojevic S, Cole TJ, Baur X, Hall GL, Culver BH, et al. Multi-ethnic reference values for spirometry for the 3-95-yr age range: The global lung function 2012 equations. Eur Respir J. 2012;40(6):1324–43.
